# An enriched environment prevents diabetes-induced cognitive impairment in rats by enhancing exosomal miR-146a secretion from endogenous bone marrow-derived mesenchymal stem cells

**DOI:** 10.1371/journal.pone.0204252

**Published:** 2018-09-21

**Authors:** Kenta Kubota, Masako Nakano, Eiji Kobayashi, Yuka Mizue, Takako Chikenji, Miho Otani, Kanna Nagaishi, Mineko Fujimiya

**Affiliations:** 1 Department of Anatomy, Sapporo Medical University, School of Medicine, Sapporo, Hokkaido, Japan; 2 Department of Physical Therapy, Hokkaido Chitose Rehabilitation University, Chitose, Hokkaido, Japan; University of Florida, UNITED STATES

## Abstract

Increasing evidence suggests that an enriched environment (EE) ameliorates cognitive impairment by promoting repair of brain damage. However, the mechanisms by which this occurs have not been determined. To address this issue, we investigated whether an EE enhanced the capability of endogenous bone marrow-derived mesenchymal stem/stromal cells (BM-MSCs) to prevent hippocampal damage due to diabetes by focusing on miRNA carried in BM-MSC-derived exosomes. In diabetic streptozotocin (STZ) rats housed in an EE (STZ/EE), cognitive impairment was significantly reduced, and both neuronal and astroglial damage in the hippocampus was alleviated compared with STZ rats housed in conventional cages (STZ/CC). BM-MSCs isolated from STZ/CC rats had functional and morphological abnormalities that were not detected in STZ/EE BM-MSCs. The miR-146a levels in exosomes in conditioned medium of cultured BM-MSCs and serum from STZ/CC rats were decreased compared with non-diabetic rats, and the level was restored in STZ/EE rats. Thus, the data suggest that increased levels of miR-146a in sera were derived from endogenous BM-MSCs in STZ/EE rats. To examine the possibility that increased miR-146a in serum may exert anti-inflammatory effects on astrocytes in diabetic rats, astrocytes transfected with miR-146a were stimulated with advanced glycation end products (AGEs) to mimic diabetic conditions. The expression of IRAK1, NF-κB, and tumor necrosis factor-α was significantly higher in AGE-stimulated astrocytes, and these factors were decreased in miR-146a-transfected astrocytes. These results suggested that EEs stimulate up-regulation of exosomal miR-146a secretion by endogenous BM-MSCs, which exerts anti-inflammatory effects on damaged astrocytes and prevents diabetes-induced cognitive impairment.

## Introduction

Cognitive impairment associated with diabetes is a worldwide problem, and diabetes increases the risk of dementia 2- to 3-fold [1]. Hyperglycemia induces brain damage by increasing arteriosclerosis, oxidative stress, and insulin resistance in the central nervous system (CNS) [2]. In type 1 diabetic rodent models, various abnormalities have been noted, including aberrant neuronal activities, decreased synaptic plasticity, and astroglial damage in the hippocampus [3, 4]. In type 2 diabetic rodent models, reduced insulin signaling and metabolic disturbance, in addition to neuronal and astroglial abnormalities in the hippocampus, have been reported [5, 6].

Cognitive rehabilitation is widely used as a non-pharmacological intervention for patients with cognitive impairment caused by diabetes, Alzheimer's disease, or Parkinson's disease [7]. However, the mechanisms that lead to memory and psychological improvement have not been fully clarified [8]. To elucidate how environmental stimulation affects cognitive impairment, we investigated the effectiveness of an enriched environment (EE) for preventing progression of cognitive impairment caused by diabetes in rodents.

In an EE, animals have frequent opportunities for social interaction and physical activity compared with conventional cages [9]. In an EE, animals are kept in large groups in a spacious cage with running wheels, toys, and mazes that are moved frequently [9]. An EE provides beneficial effects in rodent models of neurodegenerative disorders such as Alzheimer's disease and Parkinson's disease [10, 11]. An EE induces experience-dependent plasticity [12], promotes neurogenesis [13], increases the expression of neurotransmitters and trophic factors including brain-derived neurotrophic factor [14–16], enhances the density of dendritic spines [17], and drives epigenetic changes [18]. An EE stimulates circulating immune cells to secrete anti-inflammatory exosomal microRNAs (miRNAs), which may be effective against demyelinating disease in the CNS [19]. In diabetic models, an EE increases neurogenesis in the hippocampus and the density of dendrites and spines via experience-dependent plasticity [13, 20, 21]. Although evidence is accumulating that an EE improves cognitive impairment by promoting repair of brain damage, no previous studies have demonstrated a relationship between EE-enhanced brain functions and functional roles of bone marrow-derived mesenchymal stem/stromal cells (BM-MSCs).

Recently, we showed that systemic injection of BM-MSCs ameliorates diabetes-induced cognitive impairment, and confirmed that exosomes, which are extracellular vesicles containing mRNAs, miRNAs and proteins, derived from BM-MSCs are involved in the repair of damaged astrocytes and neurons in a type 1 diabetic model [22]. BM-MSCs are a powerful tool for combatting various disorders, including hepatic dysfunction [23], nephropathy [24], and wounds [25], by suppressing inflammation and repairing damaged organs [26]. However, BM-MSCs isolated from diabetic animals do not exert sufficient therapeutic functions [27]. Exosomes are released from BM-MSCs and transported to target cells to influence recipient cell function [28]. For example, exosomes derived from BM-MSCs promote angiogenesis in acute myocardial infarction in rats [29], improve renal function [30], and enhance functional recovery in a stroke model via miR-133b transfer into neurons and astrocytes [31]. Our previous study showed that BM-MSCs can improve diabetes-induced cognitive impairment by secreting exosomes [22]. Thus, we hypothesized that an EE activates endogenous BM-MSCs and that exosomes derived from activated BM-MSCs can ameliorate neuronal and astroglial damage in diabetic models.

In this study, we investigated whether an EE enhances the capability of endogenous BM-MSCs to prevent progression of hippocampal damage caused by diabetes by focusing on miRNA carried in BM-MSC-derived exosomes.

## Results

### An EE prevents learning and memory impairment in diabetic rats

**Fig 1:**
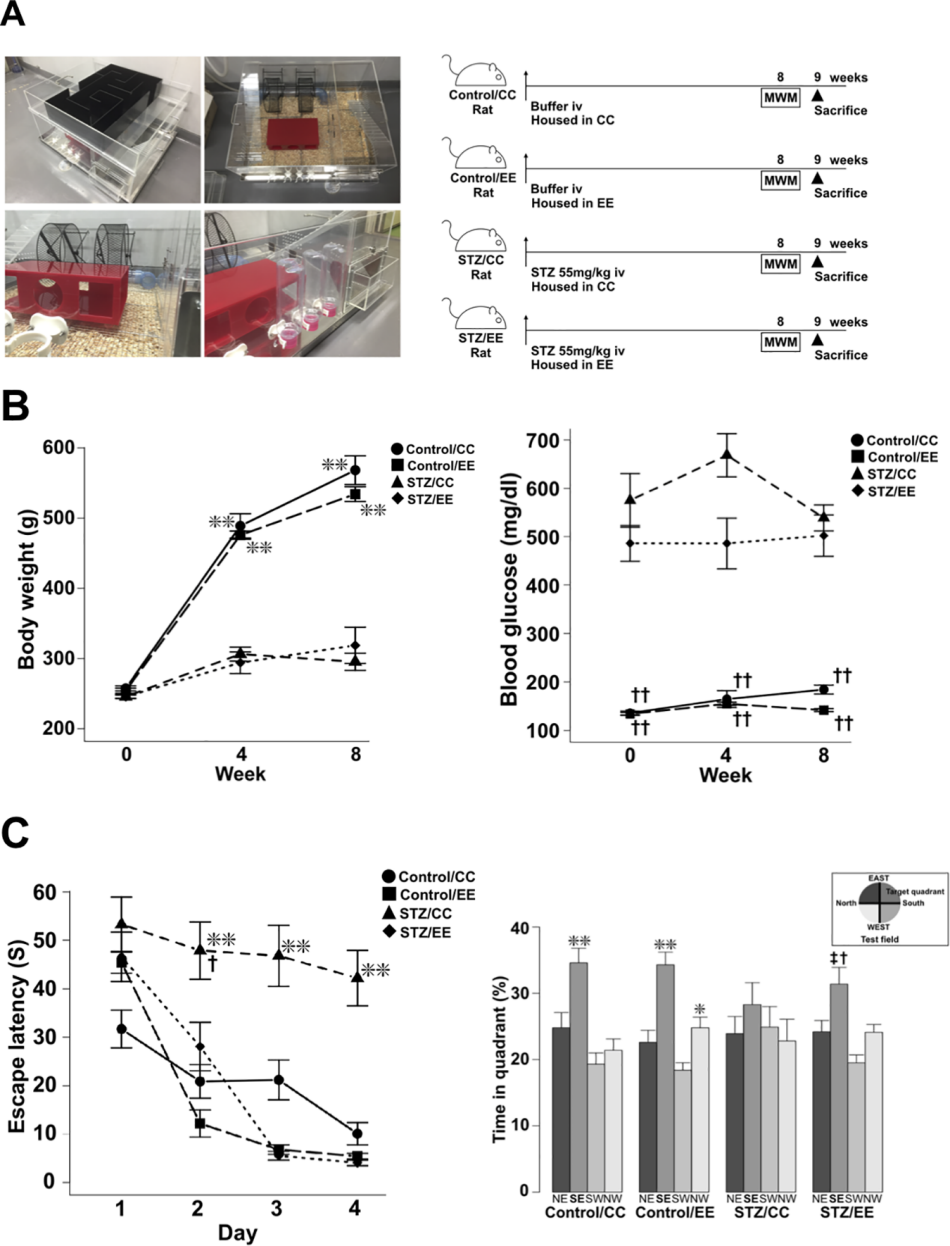
Effects of an EE on cognitive function in STZ diabetic rats. (A) EE equipment and experimental protocols. (B) Changes in body weight and serum blood glucose levels from 1 to 8 weeks after STZ injection. Body weight; **P < 0.01, Control/CC vs. STZ/CC and STZ/EE, Control/EE vs. STZ/CC and STZ/EE, Blood glucose; ^††^*P* < 0.01, Control/CC vs. STZ/CC and STZ/EE, Control/EE vs. STZ/CC and STZ/EE, two-way ANOVA, Bonferroni post-hoc test. Values are means ± standard error, n = 10-12/group. (C) The hidden platform sessions of the MWM test (left panel). Day 2; **P < 0.01, Control/CC and Control/EE vs. STZ/CC, ^†^*P* < 0.05, STZ/EE vs. STZ/CC, Days 3 and 4; **P < 0.01, Control/CC, Control/EE and STZ/EE vs. STZ/CC, two-way ANOVA, Bonferroni post-hoc test. Values are means ± standard error, n = 10–12. The probe test (right panel). Control/CC; **P < 0.01, SE vs. NE, SW, and NW, Control/EE; **P < 0.01, SE vs. NE, SW, and NW, *P < 0.05, SW vs. NW, STZ/EE; ^†^*P* < 0.05, SE vs. NE and NW, ^††^*P* < 0.01, SE vs. SW, one-way ANOVA, Bonferroni post-hoc test.

Control rats in control cages (control/CC) and streptozotocin-treated rats in control cages (STZ/CC) were housed in CCs, whereas Control/EE and STZ/EE rats were housed in EE cages for 8 weeks after STZ or vehicle injection. Subsequently, the animals underwent a series of Morris Water Maze (MWM) tests and were sacrificed for immunohistochemical analysis (Fig 1A). During the experimental period from 0 to 8 weeks after STZ injection, no differences were observed in the body weights or blood glucose levels of the STZ/CC and STZ/EE groups (Fig 1B).

In the hidden platform training of the MWM tests, STZ/CC rats took longer to reach the platform than Control/CC and Control/EE rats on Days 2, 3, and 4. On the other hand, STZ/EE rats had a shortened escape latency compared with the STZ/CC rats on Days 2, 3, and 4 (Fig 1C left panel: Day 2; *P* < 0.01, Control/CC and Control/EE vs. STZ/CC, *P* < 0.05, STZ/EE vs. STZ/CC, Days 3 and 4; *P* < 0.01, Control/CC, Control/EE, and STZ/EE vs. STZ/CC). We found no differences in the swimming speeds among the four experimental groups (Control/CC, 0.28 ± 0.01 ms^−1^; Control/EE, 0.25 ± 0.01 ms^−1^; STZ/CC, 0.25 ± 0.01 ms^−1^; STZ/EE, 0.23 ± 0.02 ms^−1^). Therefore, we concluded that no sensorimotor deficits were caused by diabetes and that the results of the MWM tests are comparable. During the probe test, in Control/CC, Control/EE, and STZ/EE rats, the time spent in the target quadrant (South East, SE) was significantly longer than in other quadrants. In contrast, in STZ/CC rats, no difference was found in the time spent in the SE target quadrant compared to other quadrants (Fig 1C right panel: Control/CC; *P* < 0.01, SE vs. NE, SW, and NW, Control/EE; *P* < 0.01, SE vs. NE, SW, and NW, *P* < 0.05, SW vs. NW, STZ/EE; *P* < 0.05, SE vs. NE and NW, *P* < 0.01, SE vs. SW). No difference was found in the time spent in the target quadrant among the four experimental groups.

### An EE prevents neuronal loss, decreases oxidative stress, and enhances synaptic density

**Fig 2:**
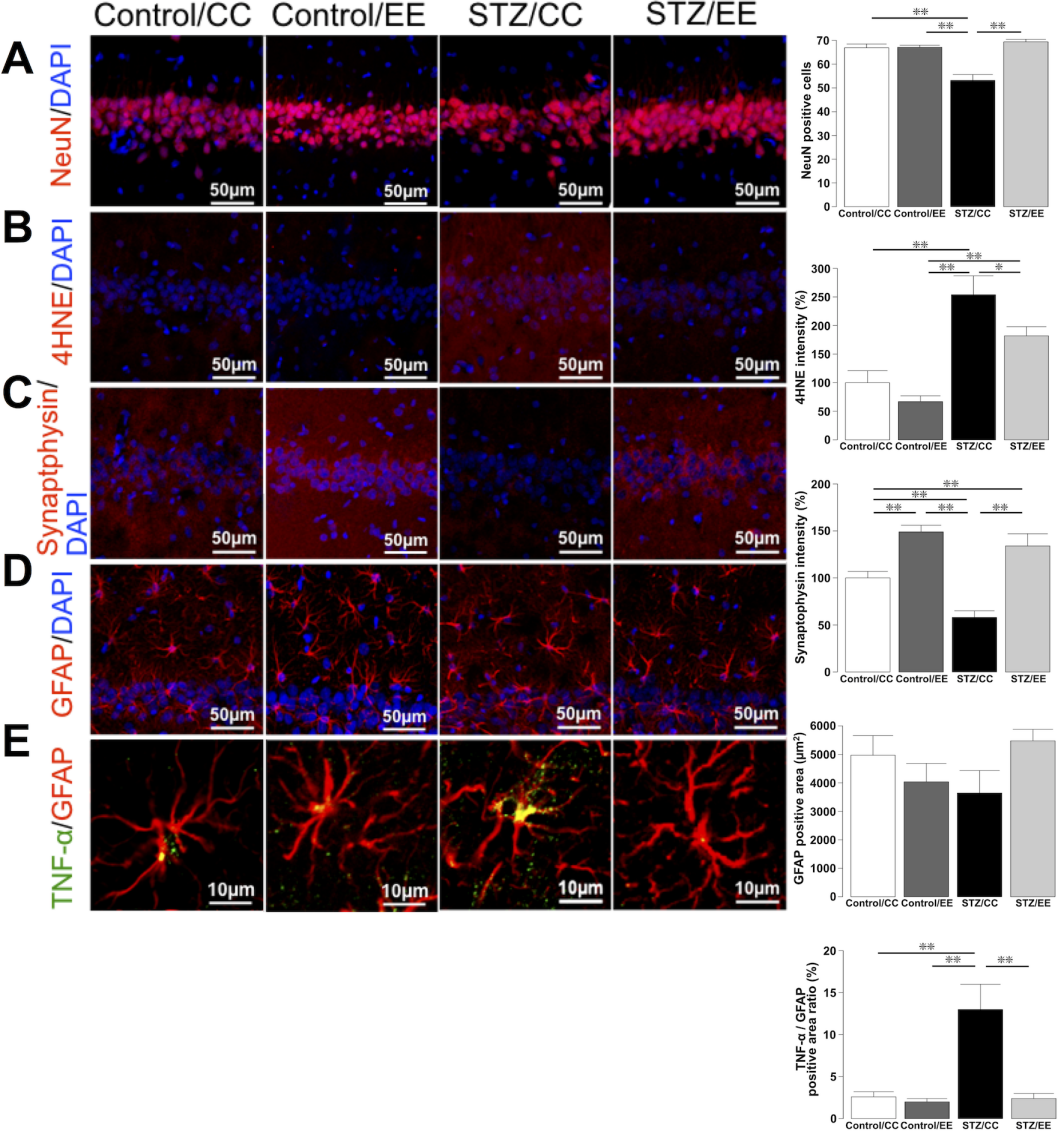
Immunohistochemical analysis of the rat hippocampal CA1 region. (A) The number of NeuN-positive cells in the hippocampal CA1 region. (B) The intensity of the 4HNE (4-hydroxynonenal)-positive area. (C) The intensity of synaptophysin staining. (D) The area of GFAP positivity. (E) The ratio of the tumor necrosis factor (TNF)-α positive area to the GFAP-positive area. (A-E) *P < 0.05, **P < 0.01, one-way ANOVA, Bonferroni post-hoc test. Values are means ± standard error, n = 6-7/group.

We investigated the mechanisms by which an EE prevents diabetes-induced cognitive impairment. The number of NeuN-positive cells in the hippocampal CA1 region in STZ/CC rats was significantly lower than that in Control/CC and Control/EE rats, whereas that of STZ/EE rats was significantly higher than that in STZ/CC rats (Fig 2A: *P* < 0.01, Control/CC, Control/EE, and STZ/EE vs. STZ/CC). The staining intensity of the 4-hydroxynonenal (4HNE)-positive area, an oxidative stress marker, was significantly increased in the CA1 region in STZ/CC rats, whereas the staining intensity in STZ/EE rats was equivalent to that of Control/CC rats (Fig 2B: *P* < 0.01, Control/CC and Control/EE vs. STZ/CC, *P* < 0.05, STZ/EE vs. STZ/CC). Furthermore, the intensity of synaptophysin staining was significantly decreased in STZ/CC rats, whereas Control/EE and STZ/EE rats had a significantly higher staining intensity than Control/CC and STZ/CC rats, respectively. (Fig 2C: *P* < 0.01, Control/CC, Control/EE, and STZ/EE vs. STZ/CC). These results suggest that an EE prevents neuronal loss, decreases oxidative stress, and enhances synaptic plasticity in the CA1 region in STZ diabetic rats.

### An EE prevents increases in astroglial inflammation

When we investigated the effects of an EE on astrocytes using immunohistochemical analysis for the astrocyte marker glial fibrillary acidic protein (GFAP), we found no significant differences in GFAP-positive areas in the CA1 region among the four groups (Fig 2D). Thus, we performed immunohistochemical overlap staining for GFAP and tumor necrosis factor (TNF)-α (an inflammatory cytokine) to examine the inflammatory changes in astrocytes. Although the ratio of the TNF-α-positive area to the GFAP-positive area was significantly increased in STZ/CC rats, the ratio in STZ/EE rats was equivalent to that in Control/CC and Control/EE rats (Fig 2E: *P* < 0.01, Control/CC and Control/EE vs. STZ/CC). These results suggest that an EE prevents cytokine expression in astrocytes in the CA1 region in diabetic rats.

### An EE prevents BM-MSC abnormalities in STZ rats

**Fig 3:**
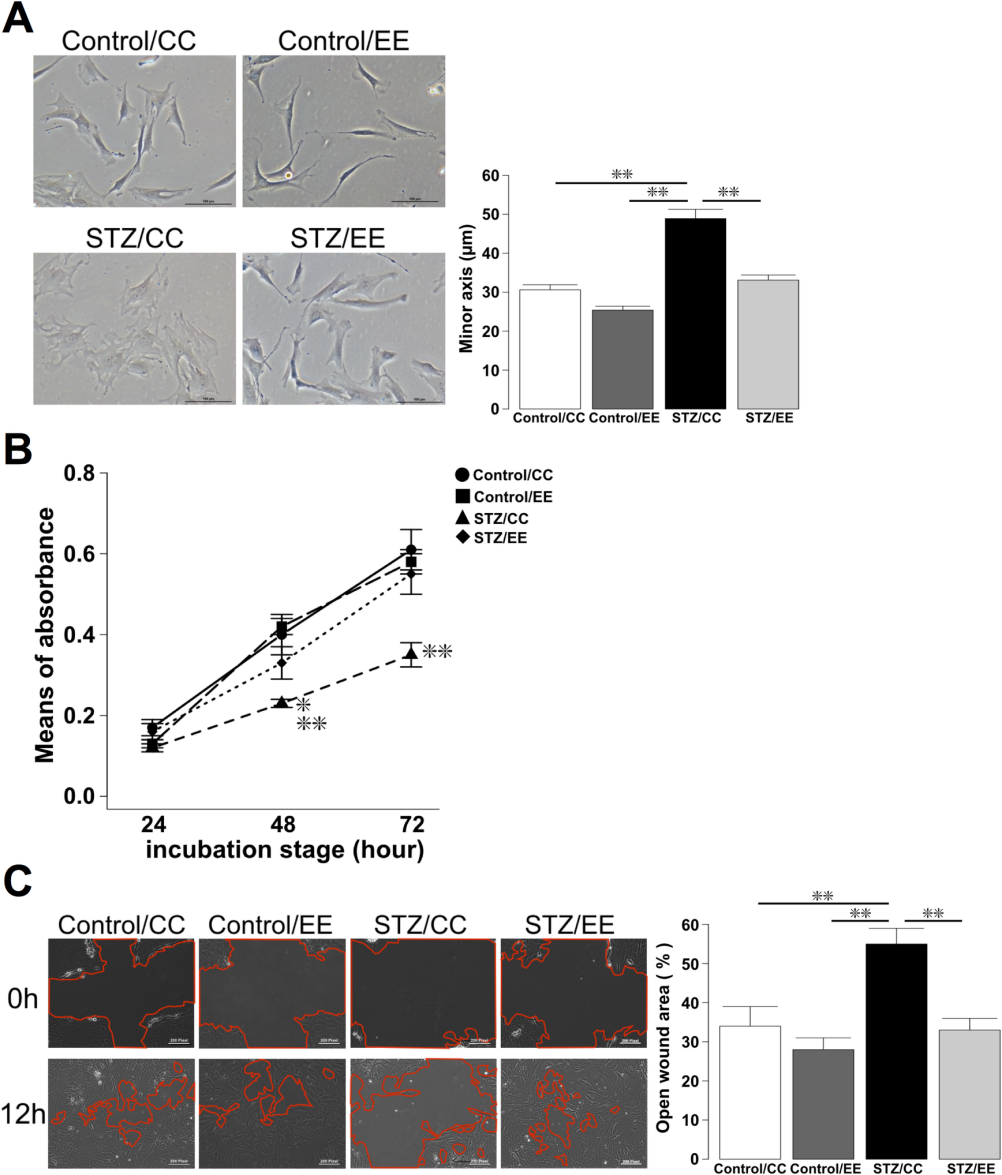
Comparison of BM-MSCs isolated from Control/CC, Control/EE, STZ/CC, and STZ/EE rats. (A) Phase-contrast microscopy analysis. **P < 0.01, one-way ANOVA, Bonferroni post-hoc test. Values are means ± standard error, n = 6/group. (B) MTT assays to analyze proliferation. 48 h; *P < 0.05, STZ/EE vs. STZ/CC, **P < 0.01, Control/CC and Control/EE vs. STZ/CC, 72 h; **P < 0.01, Control/CC, Control/EE, and STZ/EE vs. STZ/CC, two-way ANOVA, Bonferroni post-hoc test. Values are means ± standard error, n = 10-12/group. (C) Scratch assays. **P < 0.01, one-way ANOVA, Bonferroni post-hoc test. Values are means ± standard error, n = 6/group.

We investigated the characteristics of BM-MSCs isolated from the Control/CC, Control/EE, STZ/CC, and STZ/EE groups, focusing on histological findings, and proliferation and migration abilities. Phase-contrast microscopy analysis revealed that the shape of BM-MSCs from STZ/CC rats was flat and broad compared with BM-MSCs from Control/CC and Control/EE rats, in which BM-MSCs were slim and spindle-shaped with long cytoplasmic processes. On the other hand, the shapes of BM-MSCs from STZ/EE rats were similar to those in Control/CC and Control/EE rats (Fig 3A). The minor axis of the cells, defined as the minimal length passing through the nucleus, was significantly longer in BM-MSCs from STZ/CC rats, whereas the length in STZ/EE BM-MSCs was similar to that in BM-MSCs obtained from Control/CC and Control/EE rats (Fig 3A: *P* < 0.01, Control/CC and Control/EE vs. STZ/CC).

Cell growth measured by the MTT proliferation assay was reduced in STZ/CC BM-MSCs compared to BM-MSCs obtained from Control/CC and Control/EE rats at 48 and 72 h; however, proliferation of STZ/EE BM-MSCs was significantly greater than that of STZ/CC BM-MSCs at 48 and 72 h (Fig 3B: 48 h; *P* < 0.05, STZ/EE vs. STZ/CC, *P* < 0.01, Control/CC and Control/EE vs. STZ/CC, 72 h; *P* < 0.01, Control/CC, Control/EE, and STZ/EE vs. STZ/CC). Furthermore, cell mobilization assessed by the scratch assay was lower in STZ/CC BM-MSCs and higher in STZ/EE BM-MSCs. Notably, the open wound area in cultures of STZ/EE BM-MSCs was significantly smaller than that in cultures of STZ/CC BM-MSCs (Fig 3C: *P* < 0.01, Control/CC, Control/EE, and STZ/EE vs. STZ/CC). These results suggest that an EE prevents BM-MSC abnormalities caused by STZ-induced diabetes.

### Intravenous (iv) injection of BM-MSCs isolated from STZ/EE rats ameliorates learning and memory impairments in diabetic mice

**Fig 4:**
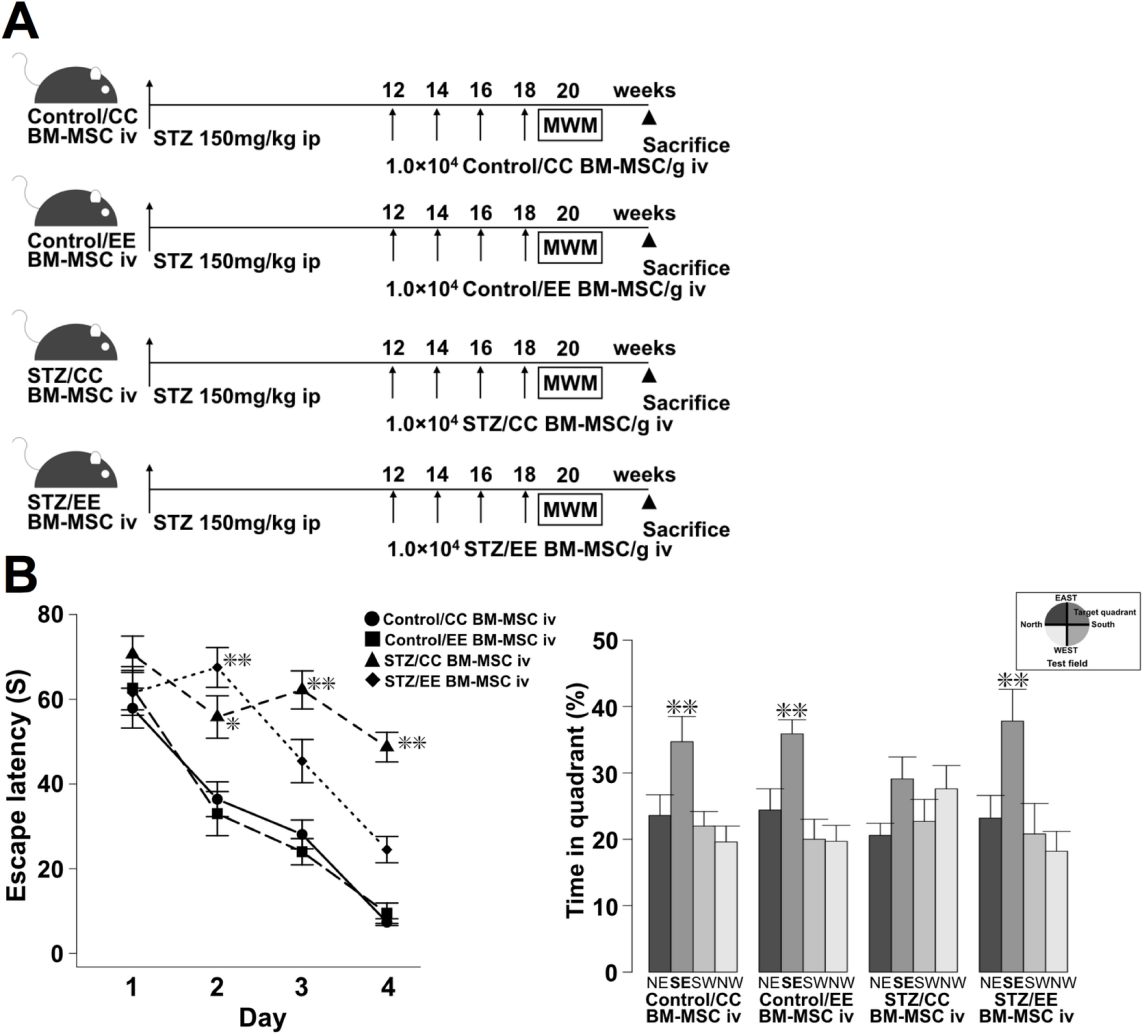
Intravenous injection of BM-MSCs isolated from Control/CC, Control/EE, STZ/CC, or STZ/EE rats into STZ-induced diabetic mice. (A) Experimental protocol. (B) The hidden platform sessions of the MWM test (left panel). Day 2; *P < 0.05, Control/CC BM-MSCs iv vs. STZ/CC BM-MSCs iv, Control/EE BM-MSCs iv vs. STZ/CC BM-MSCs iv, **P < 0.01, Control/CC BM-MSCs iv vs. STZ/EE BM-MSCs iv, Control/EE BM-MSCs iv vs. STZ/EE BM-MSCs iv, Days 3 and 4; **P < 0.01, Control/CC, Control/EE, and STZ/EE BM-MSCs iv vs. STZ/CC BM-MSCs iv, two-way ANOVA, Bonferroni post-hoc test. Values are means ± standard error, n = 10-11/group. The probe test (right panel). **P < 0.01, SE vs. NE, SW, and NW, one-way ANOVA, Bonferroni post-hoc test.

We hypothesized that an EE influences not only *in vitro* functions of BM-MSCs but also *in vivo* functions of BM-MSCs. To examine this hypothesis, we injected BM-MSCs derived from Control/CC, Control/EE, STZ/CC, and STZ/EE rats into diabetic mice. Based on our previous study, 1 × 10^4^ BM-MSCs/g body weight were administered iv from 12 weeks after STZ injections, four times at 2-week intervals [22]. The mice then underwent a series of MWM tests (Fig 4A).

At the 20th week after STZ injection, no significant differences in body weight or blood glucose levels were found among the four groups (S1 Fig). In the hidden platform training of the MWM tests, STZ/CC BM-MSC iv mice took longer to reach the platform than Control/CC and Control/EE BM-MSC iv mice on Days 2, 3, and 4. On the other hand, STZ/EE BM-MSC iv mice had a shortened escape latency compared with the STZ/CC BM-MSC iv mice on Days 3 and 4. (Fig 4B left panel: Day 2; *P* < 0.05, Control/CC BM-MSCs iv vs. STZ/CC BM-MSCs iv, Control/EE BM-MSCs iv vs. STZ/CC BM-MSCs iv, *P* < 0.01, Control/CC BM-MSCs iv vs. STZ/EE BM-MSCs iv, Control/EE BM-MSCs iv vs. STZ/EE BM-MSCs iv, Days 3 and 4; *P* < 0.01, Control/CC, Control/EE, and STZ/EE BM-MSC iv vs. STZ/CC BM-MSC iv). No significant differences were found in the swimming speed among the four groups (Control/CC BM-MSCs iv, 0.095 ± 0.002 ms^−1^; Control/EE BM-MSCs iv, 0.098 ± 0.001 ms^−1^; STZ/CC BM-MSCs iv, 0.093 ± 0.003 ms^−1^; STZ/EE BM-MSCs iv, 0.091 ± 0.001 ms^−1^). Therefore, we concluded that diabetes did not cause sensorimotor deficits and that the results of the MWM tests are comparable. During the probe test, in the STZ/CC BM-MSC iv group, no difference was found in the time spent in the SE target quadrant compared to other quadrants. In contrast, in the Control/CC, Control/EE, and STZ/EE BM-MSC iv groups, the time spent in the target quadrant (SE) was significantly longer than in other quadrants (Fig 4B right panel: Control/CC, Control/EE, and STZ/EE BM-MSCs iv; *P* < 0.01, SE vs. NE, SW, and NW). No difference was found in the time spent in the target quadrant among the four experimental groups.

### Increased exosomal miR-146a secretion was observed in the conditioned medium of cultured STZ/EE BM-MSCs and serum from STZ/EE rats

**Fig 5:**
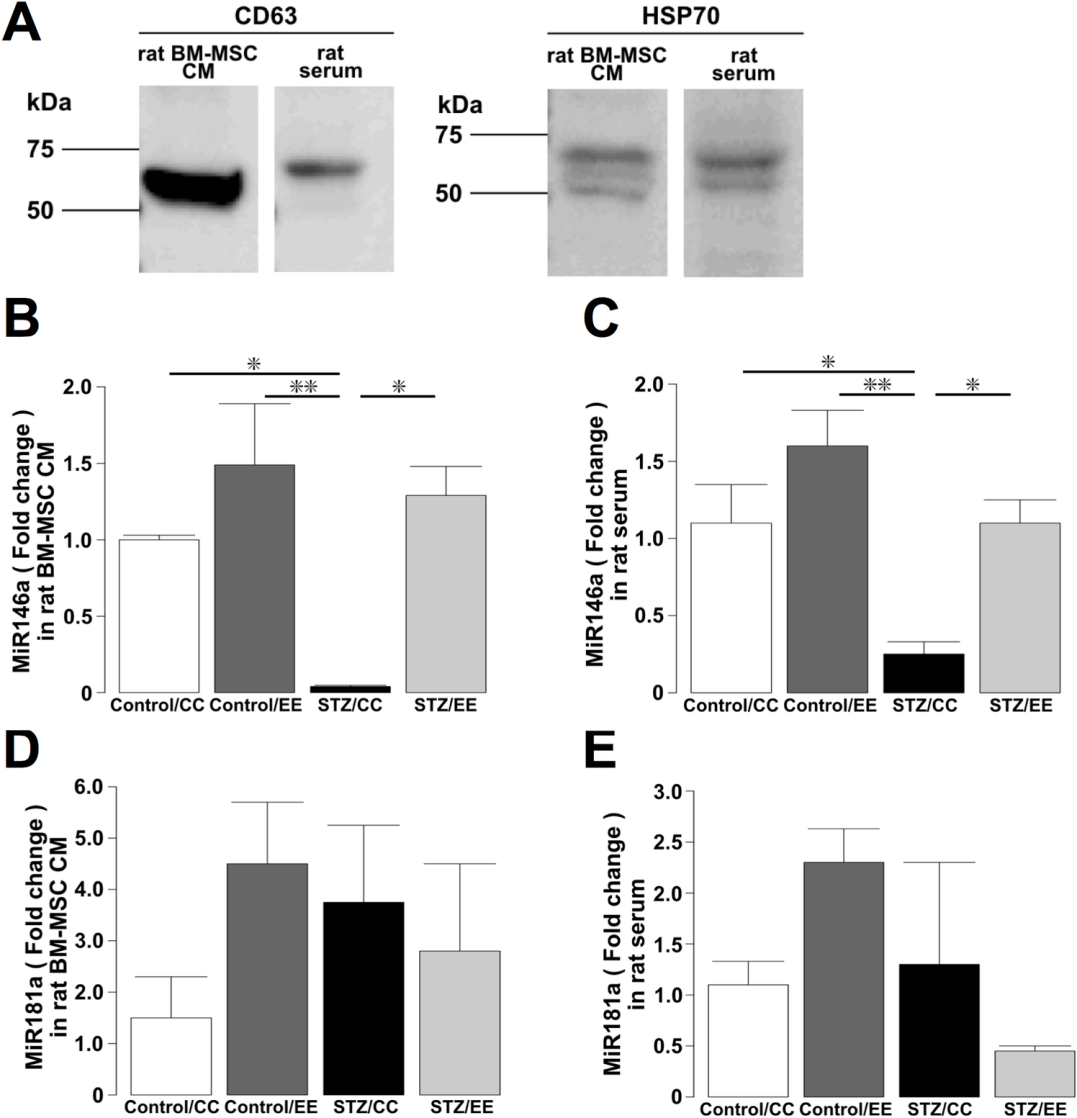
Analysis of exosomal miRNA in conditioned medium of cultured BM-MSCs and rat serum. (A) Western blotting for CD63 and HSP70 detected exosomes extracted from conditioned medium of cultured BM-MSCs or rat serum. The figure shows a cropped image of the blot. Full-length blots are presented in S2 Fig. (B) The concentration of miR-146a in exosomes extracted from conditioned medium of cultured BM-MSCs from the four groups. n = 3-4/group. (C) The serum levels of exosomal miR-146a from the four groups. n = 6-8/group. (D) The concentration of miR-181a in exosomes extracted from conditioned medium of cultured BM-MSCs from the four groups. n = 5-6/group. (E) The serum levels of exosomal miR-181a from the four groups. n = 4-5/group. *P < 0.05, **P < 0.01, one-way ANOVA, Bonferroni post-hoc test. Values are means ± standard error.

We examined the concentration of exosomal miR-146a in the conditioned medium of cultured BM-MSCs. First, we confirmed the presence of exosomes in conditioned medium of cultured BM-MSCs and rat serum by assessing the presence of the common exosomal markers CD63 and HSP70 (Fig 5A, S2 Fig). The concentration of miR-146a in exosomes released by STZ/CC BM-MSCs was significantly lower than that from Control/CC BM-MSCs, whereas the levels in Control/EE and STZ/EE BM-MSCs were significantly higher than those in STZ/CC BM-MSCs (Fig 5B: *P* < 0.05, Control/CC and STZ/EE BM-MSCs vs. STZ/CC BM-MSCs, *P* < 0.01, Control/EE BM-MSCs vs. STZ/CC BM-MSCs). The serum levels of exosomal miR-146a in STZ/CC rats were significantly lower than levels in Control/CC and Control/EE rats, whereas the serum levels of exosomal miR-146a in STZ/EE rats were significantly higher than those in STZ/CC rats (Fig 5C: *P* < 0.05, Control/CC and STZ/EE rats vs. STZ/CC rats, *P* < 0.01, Control/EE rats vs. STZ/CC rats).

We also investigated the concentration of miR-181a in conditioned medium of cultured BM-MSCs. No differences in exosomal miR-181a levels were detected in conditioned medium of cultured BM-MSCs from the four groups (Fig 5D). Moreover, no differences in the serum levels of exosomal miR-181a were detected in the four groups of rats (Fig 5E).

### Increased expression of miR-146a exerts anti-inflammatory effects on astrocytes in diabetic rats

**Fig 6:**
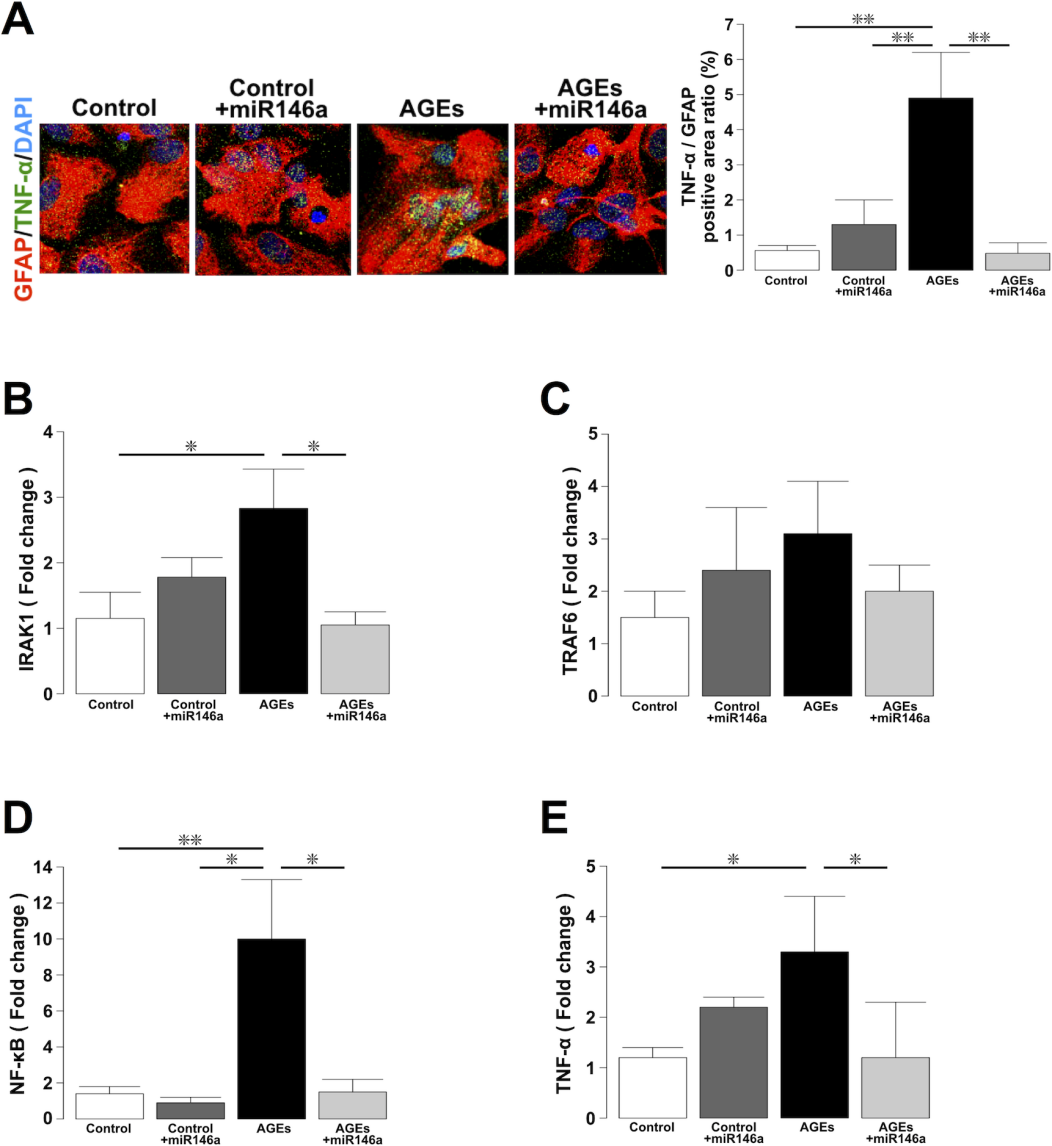
Analysis of whether increased expression of miR-146a exerts anti-inflammatory effects on astrocytes in diabetic rats. (A) Immunohistochemical analysis of the ratio of the TNF-α-positive area to the GFAP-positive area. n = 8/group. (B) The expression of IRAK1 in the four groups. n = 4-5/group. (C) The expression of TRAF6 in the four groups. n = 3-7/group. (D) The expression of NF-κB in the four groups. n = 4-7/group. (E) The expression of TNF-α in the four groups. n = 3-7/group. (A-E) *P < 0.05, **P < 0.01, one-way ANOVA, Bonferroni post-hoc test. Values are means ± standard error.

To examine whether the increased level of exosomal miR-146a in the serum exerts anti-inflammatory effects on astrocytes in this diabetes model, miR-146a was transfected into astrocytes, which were stimulated by advanced glycation end products (AGEs) to mimic diabetic conditions. First, we performed immunohistochemical overlap staining for GFAP and TNF-α. The ratio of the TNF-α-positive area to the GFAP-positive area was significantly increased in the AGEs group, whereas the ratio in the AGEs+miR-146a group was equivalent to the levels in the Control and Control+miR-146a groups (Fig 6A: *P* < 0.01, Control, Control+miR-146a and AGEs+miR-146a groups vs. the AGEs group). The expression of interleukin-1 receptor associated kinase 1 (IRAK1), an upstream regulatory inflammatory cytokine in AGEs, was significantly higher in the AGEs group than in the Control, whereas the expression of IRAK1 in the AGEs+miR-146a group was significantly lower than that in the AGEs group (Fig 6B: *P* < 0.05, Control and AGEs+miR-146a groups vs. AGEs group). However, no difference was found in the expression of tumor necrosis factor receptor-associated factor 6 (TRAF6), an upstream regulator of inflammatory cytokines, among the four groups (Fig 6C). On the other hand, the expression of NF-κB was significantly higher in the AGEs group than in the Control and Control+miR-146a groups, whereas the expression of NF-κB in the AGEs+miR-146a group was significantly lower than that in the AGEs group (Fig 6D: *P* < 0.05, Control+miR-146a and AGEs+miR-146a groups vs. AGEs group, *P* < 0.01, Control group vs. AGEs group). Moreover, the expression of TNF-α in the AGEs group was significantly higher than that in the Control, whereas that in the AGEs+miR-146a group was equivalent to that in the Control (Fig 6E: *P* < 0.05, Control and AGEs+miR-146a groups vs. AGEs group). These results suggest that increased expression of miR-146a ameliorated diabetes-induced inflammatory changes in astrocytes.

## Discussion

To our knowledge, this is the first report to show that an EE can activate BM-MSCs and prevent cognitive impairment caused by diabetes. We found that an EE inhibits diabetes-induced cognitive impairment even under hyperglycemic conditions, and at the same time prevents functional abnormalities in cultured BM-MSCs caused by diabetes. We also found that systemic injection of BM-MSCs from rats housed in an EE has therapeutic potential for the treatment of diabetes-induced cognitive impairment. Finally, we showed that exosomal miR-146a originating from endogenous BM-MSCs may be a key molecule that is up-regulated in response to an EE, and may play a role in minimizing cognitive impairment by reducing astrocytic inflammation.

We housed diabetic rats in an EE for 8 weeks after STZ injection and then conducted MWM tests to examine learning and memory abilities. STZ/CC rats showed abnormalities in both learning and memory; however, these abnormalities were restored in STZ/EE rats to the level of Control/CC and Control/EE rats. Because no difference was found in the swimming speeds, body weights, or blood glucose levels between STZ/CC and STZ/EE rats, the results suggest that prevention of cognitive decline by an EE may not be due to reduced hyperglycemia or sensorimotor deficits.

Immunohistochemical analysis of the hippocampus revealed that STZ/EE rats had reduced oxidative stress, increased synaptic density, and down-regulated TNF-α expression in astrocytes. In the present study, the GFAP-positive area was not altered among the four groups. On the other hand, previous reports have shown an increased number of GFAP-positive cells [32] or decreased GFAP immunoreactivity [32, 33] in the brains of diabetic models. Therefore, we further analyzed astrocytes by examining co-expression of TNF-α and GFAP, because TNF-α is a useful marker of astrocyte inflammation [34]. The results showed that TNF-α expression in astrocytes was increased in diabetic rats, whereas an EE prevented this phenomenon. Astrocytes supply lactate to neurons, adjust extracellular K^+^ levels, remove excess glutamate, and promote synaptogenesis [35, 36]. However, TNF-α in astrocytes induces generation of excessive reactive oxygen species and leads to neuronal death [37, 38]. Therefore suppression of TNF-α expression in astrocytes by an EE may prevent neuronal loss, stimulate synaptogenesis, and subsequently maintain cognitive function.

Recently, our group reported that systemic injection of BM-MSCs ameliorates diabetic complications, including nephropathy, hepatic dysfunction, and cognitive impairment without improving hyperglycemia [22–24]. In addition, BM-MSCs suppress oxidative stress, reverse astrocyte abnormalities, and facilitate synaptic plasticity in hippocampi damaged by diabetes [22]. BM-MSCs also improve neuronal recovery after stroke by secreting neurotrophic factors, increasing angiogenesis and synaptogenesis, and promoting glial remodeling [39]. In the present study, we found that an EE prevents hippocampal damage caused by diabetes in a similar way as systemic injection of BM-MSCs [22]. A recent report showed that endogenous BM-MSCs stimulated with granulocyte colony stimulating factor increase the regeneration of damaged inner ear hair cells without injection of exogenous BM-MSCs [40]. Thus, we hypothesized that an EE has the potential to prevent cognitive impairment at least in part by activating endogenous BM-MSCs. Thus, we investigated how an EE influences endogenous BM-MSC function, and how EE-activated BM-MSCs prevent cognitive impairment in diabetic animals.

In *in vitro* experiments, STZ/CC BM-MSCs exhibited an abnormal flattened shape with an increased minor axis length, impaired proliferation ability, and decreased migratory potential. These abnormalities were considered to be due to cell senescence caused by diabetes, because diabetes induces BM-MSC senescence by up-regulating autophagy [41], and aging causes enlargement of BM-MSCs [42]. Senescence of BM-MSCs reduces their proliferative ability and impairs their migration response by activating the p53/p21 pathway and inhibiting the AP-1 pathway [43]. In the present study, the abnormalities in BM-MSCs caused by diabetes were completely prevented by an EE. How this occurs is not clear at present. However, an EE may suppress the hypothalamic-pituitary-adrenal axis [20], and the subsequent reduction in cortisol levels, which are linked to cytotoxicity [44], may lead to functional enhancement of endogenous BM-MSCs.

In our previous study, systemic injection of BM-MSCs obtained from normal rats ameliorated cognitive impairment and helped repair hippocampal damage in STZ diabetic mice [22]. With this in mind, we examined the effects of BM-MSCs from Control/CC, Control/EE, STZ/CC, and STZ/EE rats on cognitive impairment in STZ mice. Based on our previous study [22], we injected BM-MSCs into mice four times at 2-week intervals 12 weeks after STZ injections, which is the time when animals show cognitive impairment. STZ/CC BM-MSC-injected mice showed impaired performance in the MWM test, whereas cognitive function was improved in Control/CC BM-MSC-, Control/EE BM-MSC-, and STZ/EE BM-MSC-injected mice.

Our results showed that diabetes induces BM-MSC abnormalities that can be reversed by an EE. Furthermore, EE-enhanced BM-MSCs have the potential to prevent cognitive impairment and hippocampal damage caused by diabetes. Because BM-MSCs release exosomes that are taken up by astrocytes and that exert neuroprotective functions [22], EE-enhanced BM-MSCs may release exosomes that contain neuroprotective components.

miRNA-146a exerts anti-inflammatory effects by suppression of TRAF6 and IRAK1, which are activators of the NF-κB pathway [45]. miR-146a expression is decreased in the hippocampus of diabetic rats, whereas the expression of IRAK1, TRAF6, and NF-κB is increased [46]. miR-146a transfection ameliorates the inflammation of astrocytes caused by exposure to interleukin (IL)-1β by inhibiting the production of cytokines including IL-6, IL-8, granulocyte colony stimulating factor, interferon-γ, interferon-γ induced protein 10 (IP-10), macrophage inflammatory protein 1-b (MIP-1b), and TNF-α [47]. Moreover, in diabetes, decreased levels of serum miR-146a correspond to chronic inflammation [48], and miR-146a deficiency leads to diabetic nephropathy by activating macrophages [49]. Hyperglycemia downregulates miR-146a expression in dorsal root ganglia neurons, and transfection of dorsal root ganglia neurons with miR-146a increases neuronal survival, even in high glucose conditions [50]. In addition, BM-MSCs improve diabetic wound healing by increasing miR-146a levels [25], and anti-inflammatory effects of miR-146a derived from umbilical cord MSCs have also been demonstrated [51].

We examined the concentrations of miR-146a in conditioned medium of cultured BM-MSCs from Control/CC, Control/EE, STZ/CC, and STZ/EE rats. The miR-146a level was decreased in STZ/CC BM-MSCs, whereas the miR-146a level in STZ/EE BM-MSCs was not different compared to Control/CC and Control/EE BM-MSCs. These results suggest that the EE prevented the decrease in miR-146a levels in exosomes derived from BM-MSCs in diabetes and maintained normal levels. Next, to examine the involvement of exosomal miR-146a derived from endogenous BM-MSCs in repairing hippocampal damage, we measured the miR-146a levels in exosomes from Control/CC, Control/EE, STZ/CC, and STZ/EE rat serum. We detected a recovery in miR-146a levels in exosomes from STZ/EE rat serum compared with STZ/CC rat serum, suggesting that increased levels of miR-146a may be derived from endogenous BM-MSCs. Although various cell types, including immune cells, release miR-146a [52], our results raise the possibility that exosomal miR-146a derived from BM-MSCs may contribute to repair of hippocampal damage caused by diabetes and subsequently to recovery of cognitive impairment.

The involvement of another miRNA, miR-181a, which exerts anti-inflammatory effects on astrocytes [53], was also examined. No differences were found in the levels of exosomal miR-181a in the conditioned medium of cultured BM-MSCs from STZ/CC and STZ/EE rats, and no differences were found in the serum levels of exosomal miR-181a in STZ/CC and STZ/EE rats, suggesting that miR-181a is not directly involved in EE-induced activation of BM-MSCs.

To confirm the possibility that increased levels of exosomal miR-146a in the serum may exert anti-inflammatory effects on astrocytes in diabetes, miR-146a was transfected into astrocytes, which were stimulated by AGEs to mimic diabetic conditions. AGEs are important mediators of chronic inflammation and contribute to the pathogenesis of diabetic complications [54]. In previous reports, AGEs were used to stimulate astrocytes to induce reactive oxygen species production and release inflammatory cytokines such as TNF-α. Therefore, these stimulated astrocytes were considered an *in vitro* model of diabetic complications [55].

In the present study, the expression of TNF-α in cultured astrocytes was significantly higher in the AGEs group, whereas that in the AGEs+miR-146a group was equivalent to the levels in the Control and Control+miR-146a groups. The expression of IRAK1, TRAF6, and NF-κB is increased in the hippocampus of diabetic rats [46], and inhibition of IRAK1 leads to reduction in NF-κB expression and decreased TNF-α production [56]. Because similar results were obtained in our results, we conclude that miR-146a may contribute to down-regulation of IRAK1 expression and subsequent reduction of NF-κB expression and TNF-α production.

Considering the effects of miR-146a *in vitro*, the increased levels of miR-146a in exosomes from serum in EE-housed rats may prevent astrocytic damage by internalizing exosomes into astrocytes, because exosomes derived from BM-MSCs are taken up by astrocytes after intracerebroventricular injection [22]. BM-MSC-derived exosomes are also taken up by neurons to some extent, and therefore, miR-146a may maintain neuronal survival in STZ/EE rats. Although many mediators beside miR-146a are secreted by BM-MSCs [57], we consider that miR-146a is a key factor that influences astrocyte functions and subsequently enhances neuroprotective effects.

In summary, our data indicate that environmental stimulation, including active communication, stress reduction, and exercise, are important for improving cognitive impairment in diabetic rodents. A deeper understanding of the precise mechanisms by which this occurs may have important ramifications for the development of more effective treatments for human patients with diabetic cognitive impairment.

## Materials and methods

### Animals

All methods for animal experiments were performed in accordance with the relevant guidelines and regulations of the Animal Experiment Committee of Sapporo Medical University (Sapporo, Japan). All experimental protocols and studies were approved by the Animal Experiment Committee of Sapporo Medical University (approval #14–127, #16–094, #17–065). Hyperglycemia was induced in 9-week-old male SD rats (Japan SLC, Shizuoka, Japan) by a single iv injection of STZ (55 mg/kg; Wako, Osaka, Japan) dissolved in citrate buffer (pH 4.5). Housing room temperature was maintained between 21–24°C, and humidity between 50–70%, with a 12:12-h light/dark cycle. All animals were allowed free access to chow and water. After confirming that blood glucose levels were above 300 mg/dL, STZ rats were divided randomly into two groups. One group was housed in conventional cages (STZ/CC), and another group was housed in EE cages (STZ/EE) for 8 weeks. Control rats were administered citrate buffer (iv). Control rats were divided randomly into two groups. One group was housed in conventional cages (Control/CC), and another group was housed in EE cages (Control/EE) for 8 weeks. Hyperglycemia was induced in male C57BL/6j mice (Japan SLC) by a single intraperitoneal (ip) injection of STZ (150 mg/kg) dissolved in citrate buffer (pH 4.5) at 13 weeks of age. We used STZ mice with blood glucose levels above 300 mg/dL.

All efforts were made to minimize pain and distress. Invasive procedures were carried out under isoflurane inhalation, and anesthetic adequacy was checked by lack of a withdrawal response to a deep toe pinch. At the end of the study, all animals were euthanized by ip injection of an overdose of sodium pentobarbital (>120 mg/kg) or inhalation of excess isoflurane. After detection of asystole, animals were exsanguinated by transcardial puncture, and all blood was collected.

### Housing conditions

During the experimental period, rats were housed in (1) conventional cages (CC, 260 mm × 420 mm × 200 mm) with two rats per cage or (2) environmental enrichment cages (EE, 600 mm × 80 0 mm × 480 mm) with 11 rats per cage and two levels according to a previous report [58]. The first level included two running wheels, one tunnel, a red rectangular house, and places for access to food and water, whereas the second level contained a maze (Fig 1A), which was changed three times a week.

### MWM test

The MWM test was carried out according to a previous study [22]. Rats and mice were placed in a maze that consisted of a circular pool (1.2 m diameter) filled with water (25 ± 1°C). This pool size has been used for both rats and mice in previous reports [59, 60]. In this experiment, animals were trained with visible platform sessions (Day 0), and then hidden platform sessions were performed four times a day at 1-h intervals (Days 1–4). The platform was in the center of the SE quadrant and was placed 1 cm below the surface of the water during the visible and hidden tests. Each trial had a limit of 90 sec, and the time to reach the platform (escape latency) and swim speed were recorded. When animals failed to reach the platform within 90 sec, they were placed on the platform for 15 sec. At Day 5, after removing the platform, the probe test was conducted in which the time spent in each quadrant during 90 sec was recorded. An automated tracking system (Any-maze, Stoelting, Wood Dale, IL, USA) was used to record the data.

### Immunohistochemical analysis

After the end of the MWM, rats were euthanized by ip injection of sodium pentobarbital (>120 mg/kg). Brains were removed from the skull and fixed in a solution of 4% paraformaldehyde and 0.2% picric acid in 0.1 M phosphate buffer for 24 h and then immersed in a 15% sucrose solution. Brains (n = 6-7/group) were cut into 20-μm thick frozen sections, and three coronal sections of the left hippocampus (2.5–3.5 mm posterior to the bregma) were chosen for immunohistochemistry.

The sections were incubated overnight at 4°C with primary antibodies against NeuN (rabbit polyclonal, 1:1000; Millipore, Darmstadt, Germany), 4HNE (rabbit polyclonal, 1:100; Abcam, Cambridge, UK), synaptophysin (rabbit polyclonal, 1:500; Sigma-Aldrich, St. Louis, MO, USA), GFAP (chicken polyclonal, 1:500; Millipore), or TNF-α (rabbit polyclonal, 1:100; Abcam). After washing, the sections were incubated with the corresponding secondary antibodies: Cy3-labeled anti-rabbit IgG (1:500; Jackson ImmunoResearch, West Grove, PA, USA) and FITC-labeled anti-chicken IgG (1:500; Millipore) for 2 h at room temperature. DAPI (Dojindo, Kumamoto, Japan) was used for nuclear staining, and images were obtained using confocal laser scanning microscopy (Nikon A1, Tokyo, Japan).

For quantitative comparisons, the number of NeuN-positive cells and the areas of GFAP positivity were evaluated in a total of six different fields per animal (two fields of 200 × 200 μm per section). The intensities of 4HNE and synaptophysin staining were measured in a total of 15 different fields per animal (five fields of 50 × 50 μm per section), and the ratio of the TNF-α-positive area to the GFAP-positive area was analyzed for a total of nine different fields per animal (three fields of 50 × 50 μm per section). All quantitative measurements were performed using Nikon NIS Elements AR software.

### Isolation of BM-MSCs

Isolation of BM-MSCs was performed according to a previous study [23]. In brief, after ip injection of sodium pentobarbital (>120 mg/kg) and confirmation of asystole, the bilateral femurs and tibias were removed, and the bone marrow was flushed out using α-MEM medium (Gibco, Life Technology Japan, Tokyo, Japan) supplemented with 15% fetal bovine serum (CCB, Nichirei Bioscience, Tokyo, Japan) and 1% penicillin/streptomycin (Pen Strep, Life Technologies, Carlsbad, CA, USA), after removal of cancellous bone. The collected cells were incubated in 15-cm dishes with 20 mL α-MEM culture medium, and maintained at 37°C in 5% CO_2_. The culture medium was changed twice a week, and the cultivated BM-MSCs were used for further analysis. We previously confirmed that isolated BM-MSCs expressed the CD90 marker but not CD45 and CD11b, and were capable of differentiating into osteoblasts, adipocytes, and chondrocyte-like cells [23].

### BM-MSC phase-contrast microscopy

Morphological images of BM-MSCs were obtained by phase-contrast microscopy (Eclipse TE200; Nikon). To evaluate the length of the minor axis, Image J software [61] was used to measure the smallest length passing through the nucleus orthogonal to the long axis. For quantitative analyses of the minor axis, all cells in five randomly selected fields at 20× magnification were counted for each rat (n = 6/group).

### BM-MSC proliferation assays

The number of cells was indirectly analyzed using Cell Counting Kit-8 (CCK-8; Dojindo). Using 96-well culture plates (Corning Costar, Sigma-Aldrich), 3.0 × 10^3^ BM-MSCs were seeded in three wells for each sample. After 24, 48, and 72 h of incubation, WST-8 solution was added, and cells were cultured for a further 2 h. The absorbance was measured with a microplate reader (Infinite M1000 Pro, TECAN, Mannedorf, Switzerland) at 490 nm (n = 10-12/group).

### Scratch assay

Cell migration into the wound area was measured after scratching a confluent layer of BM-MSCs in a 35-mm dish. The BM-MSC monolayer was scratched with a pipette tip to create a wound in a cross shape, and cells were cultured for a further 12 h. After scratching, phase-contrast time-lapse images were obtained by Axio Observer Z1 (Carl Zeiss, Oberkochen, Germany) using an enclosed incubation system (TOKAI HIT, Fujinomiya, Japan). The ratio of the open area at 12 h to the open area at 0 h was analyzed at five points per sample using Image J software [61] (n = 6/group).

### Intravenous injection of BM-MSCs

At 12 weeks after STZ ip injections of mice, they were divided randomly into four groups, which were given injections of Control/CC BM-MSCs, Control/EE BM-MSCs, STZ/CC BM-MSCs, or STZ/EE BM-MSCs (1 × 10^4^/g body weight) four times via the tail vein under isoflurane inhalation, at 2-week intervals. We pooled three BM-MSC samples closest to the median in each group according to the MTT assay, and mixed them evenly before iv injection (n = 10/group).

### Isolation of exosomes

Exosomes were isolated from conditioned medium of cultured BM-MSCs or rat serum using the methodology described in a previous report [62]. Briefly, when BM-MSCs reached 60–80% confluency in 15-cm dishes, the conventional medium was replaced with medium containing exosome-depleted fetal bovine serum (EXO-FBS-50 A-1 System Biosciences, Mountain View, CA, USA). After incubation for an additional 48 h, the total culture medium (20 mL) was collected, and the numbers of BM-MSCs were counted. The culture medium was centrifuged at 3,000 ×*g* for 15 min, and the supernatant volume corresponding to 2.0 × 10^6^ BM-MSCs was mixed with ExoQuick-TC (5:1) (System Biosciences). For example, for 4.0 × 10^6^ BM-MSCs, 10 mL (the volume corresponding to 2.0 × 10^6^ cells) was mixed with 2 mL ExoQuick-TC. For the isolation of exosomes from rat serum, ExoQuick (System Biosciences) was used according to the manufacturer's protocol. Briefly, 250 μL rat serum was mixed with 63 μL ExoQuick solution. After incubating overnight at 4°C, the mixture was centrifuged at 1,500 ×*g* for 30 min. The resulting exosome pellets were immediately used for RNA isolation or western blotting.

### Western blot analysis

Denatured proteins from exosomal pellets were separated on 12% sodium dodecyl sulfate-polyacrylamide gels and transferred to poly vinylidene di-fluoride (PVDF) membranes. After blocking with 5% skim milk, the membranes were incubated overnight at 4°C with primary antibodies against CD63 (rabbit polyclonal, 1:1,000, System Biosciences) or HSP70 (rabbit polyclonal, 1:1,000, System Biosciences). After washing and incubation with secondary horseradish peroxidase-conjugated goat anti-rabbit IgG (1:20,000, System Biosciences), the blots were developed using a Pierce western blotting substrate kit (Thermo Fisher Scientific, Waltham, MA, USA). Digital images were produced using a Las-3000 imaging system (Fujifilm Life Science, Kanagawa, Japan).

### miRNA isolation and quantitation

Total miRNAs were extracted from isolated exosomes using a mirVana PARIS Kit (Thermo Fisher Scientific), and 2 μL of 2 nM exogenous synthetic cel-miR-39 was used as an external control to normalize miRNA levels. Following RNA isolation, a TaqMan Advanced miRNA cDNA Synthesis kit (Thermo Fisher Scientific) was used for reverse transcription, and real-time PCR was carried out using the TaqMan Fast Advanced Master Mix (Thermo Fisher Scientific). The target sequences were: miR-146a: 5′-UGAGAACUGAAUUCCAUGGGUU-3′, miR-181a: 5′-AACAUUCAACGCUGUCGGUGAGU-3′, cel-miR 39: 5′-UCACCGGGUGUAAAUCAGCUUG-3′. The relative quantities of miR-146a and miR-181a were assessed by the comparative Ct method (2^−ΔΔCt^).

### Isolation and culture of primary rat astrocytes

Rat astrocytes were isolated from the hippocampal tissue of 1- to 2-day-old postnatal SD rat pups after euthanasia by excessive inhalation of isoflurane as described previously [63]. Briefly, cells were seeded at density of 1.5 × 10^5^ cells/cm^2^ in 6-cm dishes coated with poly-l-lysine (Sigma) and maintained in a 5% CO_2_ incubator at 37°C. Cells were cultured in DMEM/F-12 medium (Sigma) supplemented with 10% fetal bovine serum (CCB) and 1% penicillin/streptomycin (Life Technologies). After 24 h, the culture medium was replaced, and the medium was subsequently changed twice a week. After 7–8 days, the culture dishes were shaken at 200 rpm overnight, and then cells were trypsinized and seeded onto poly-l-lysine-coated 24-well plates or 8-well chamber slides (0.8 × 10^5^/well in 24-well plates for RNA isolation and PCR, or 0.1 × 10^5^/well in 8-well chamber slides for immunocytochemistry). The cultured cells contained more than 95% astrocytes.

### Treatment and transfection of cells

Astrocytes were transfected with 30 nM miRNA-146a mimics (QIAGEN, Hilden, Germany) using HiPerFect transfection reagent (QIAGEN), according to the manufacturer's instructions. After 24 h, AGEs (Millipore) were added at 400 mg/L and incubated for 72 h. Then, cells were washed with phosphate-buffered saline, and RNA was extracted using TRI Reagent (Molecular Research Center, Cincinnati, OH, USA) or fixed in a solution of 4% paraformaldehyde in 0.1 M phosphate buffer for 1 h at 4°C for immunocytochemistry.

### Immunohistochemical analysis for cultured astrocytes

The slide chambers were incubated overnight at 4°C with primary antibodies. The antibodies and dilutions for GFAP and TNF-α were the same as described above. After washing, the chambers were incubated with the corresponding secondary antibodies for 2 h at room temperature. DAPI (Dojindo) was used for nuclear staining, and images were obtained using confocal laser scanning microscopy (Nikon A1). The ratio of the TNF-α-positive area to the GFAP-positive area was analyzed within a total of 24 different cells/group.

### Quantitative real-time PCR

After RNA isolation, cDNA synthesis was conducted using an Omniscript RT Kit (QIAGEN). Real-time PCR was performed using SYBR green (Thermo Fisher Scientific). Sequences of primers were: NF-κB forward: 5′-AATTGCCCCGGCAT-3′, NF-κB reverse: 5′-TCCCGTAACCGCGTA-3′, IRAK1 forward: 5′-GCTGTGGACACCGAT-3′, IRAK1 reverse: 5′-GCTACACCCATCCACA-3′, TRAF6 forward: 5′-CAGTCCCCTGCACATT-3′, TRAF6 reverse: 5′-GAGGAGGCATCGCAT-3′, TNF-α forward: 5′-GTGATCGGTCCCAACAAG-3′, TNF-α reverse: 5′-AGGGTCTGGGCCATGGAA-3′, GAPDH forward: 5′-ACCACAGTCCATGCCATCAC-3′, GAPDH reverse: 5′-TCCACCACCCTGTTGCTGTA-3′. The relative quantities were assessed by the comparative Ct method (2^−ΔΔCt^).

### Statistical analysis

Data were expressed as means ± standard error (SE). Statistical analysis was performed using R software (version 3.3.2) with one-way analysis of variance (ANOVA), followed by Bonferroni post-hoc analysis. When interactions between two factors were considered, two-way ANOVA with a Bonferroni post-hoc test was performed for analysis. The difference was considered statistically significant when *P* ≤ 0.05.

## Supporting information

S1 Fig Intravenous injection of BM-MSCs isolated from Control/CC, Control/EE, STZ/CC, or STZ/EE rats into STZ-induced diabetic mice. (A) No significant changes were detected in body weight or blood glucose levels among the four groups at the 20th week after STZ injection. One-way ANOVA. Values are means ± SE, n = 10–11.
(TIFF)

S2 Fig Analysis of exosomal miRNA in conditioned medium of cultrured BM-MSCs and rat sera. (A) The full-length blots of the cropped images shown in Fig 6A. CD63 was detected in exosomes derived from conditioned medium of cultured rat BM-MSCs (Lane 1) and exosomes derived from rat serum (Lane 2). The molecular weight of CD63 is ~53 kDa. (B) The full-length blots of the cropped images shown in Fig 6A. HSP70 was detected in exosomes derived from conditioned medium of cultured rat BM-MSCs (Lane 1) and exosomes derived from rat serum (Lane 2). The molecular weight of HSP70 is 53–70 kDa.
(TIFF)
