# Supplementary figures and images for: An enriched environment prevents diabetes-induced cognitive impairment in rats by enhancing exosomal miR-146a secretion from endogenous bone marrow-derived mesenchymal stem cells
Source: PLoS One. 2018 Sep 21;13(9):e0204252. doi: 10.1371/journal.pone.0204252 (PMC6150479; doi:10.1371/journal.pone.0204252)

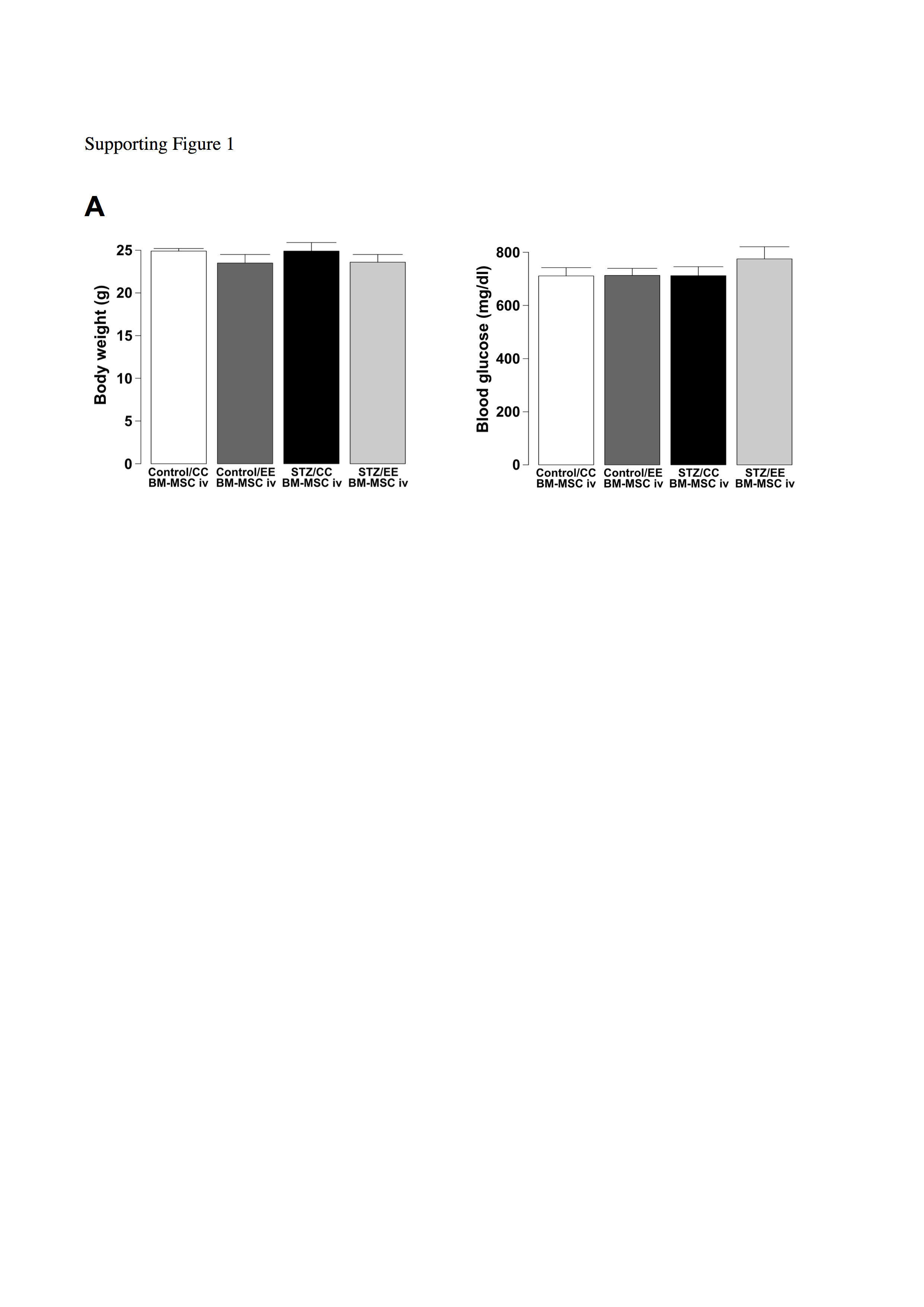

Supplement: S1 Fig — (A) No significant changes were detected in body weight or blood glucose levels among the four groups at the 20th week after STZ injection. One-way ANOVA. Values are means ± SE, n = 10–11. (TIFF) [file pone.0204252.s001.tiff]

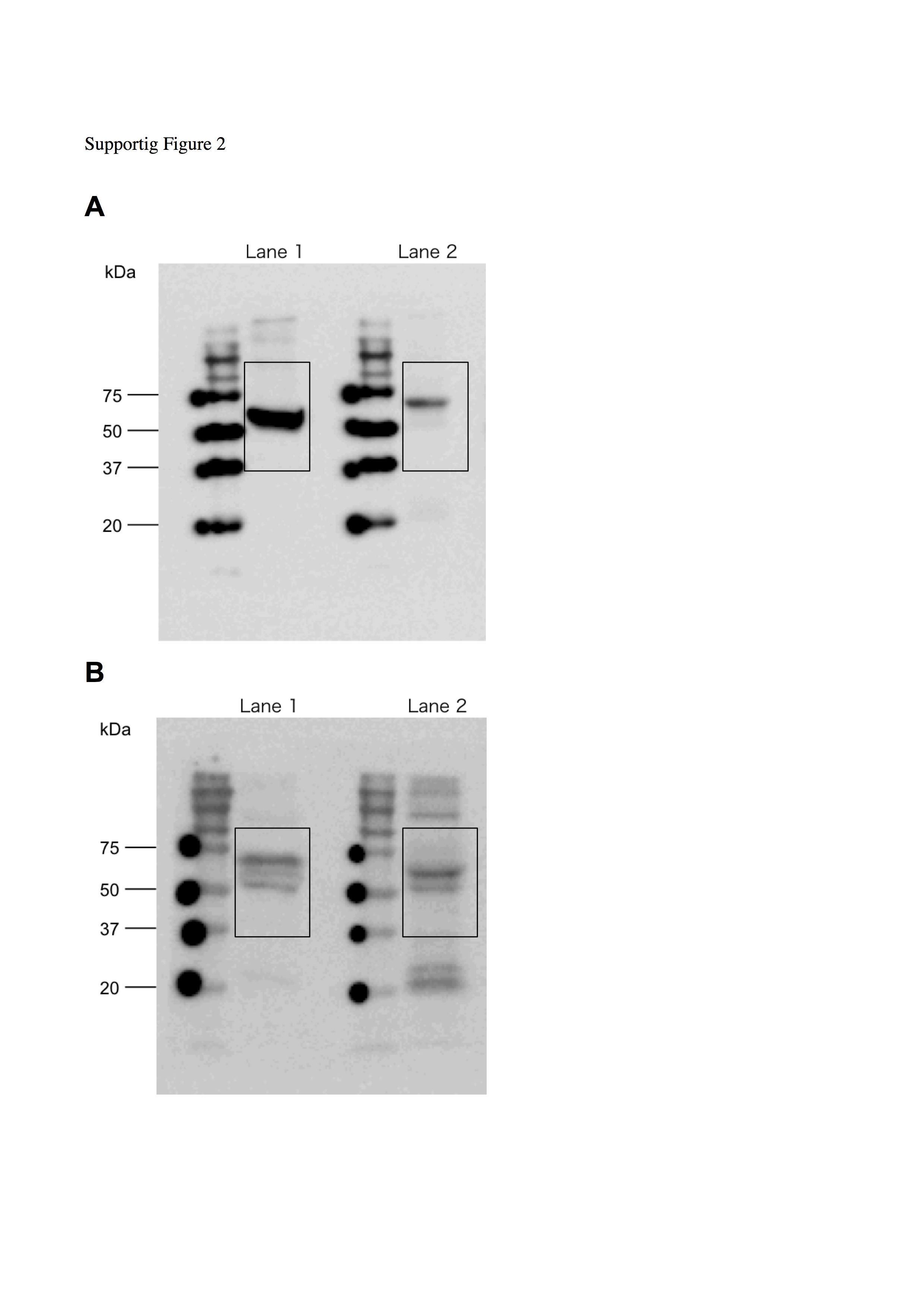

Supplement: S2 Fig — (A) The full-length blots of the cropped images shown in Fig 6A. CD63 was detected in exosomes derived from conditioned medium of cultured rat BM-MSCs (Lane 1) and exosomes derived from rat serum (Lane 2). The molecular weight of CD63 is ~53 kDa. (B) The full-length blots of the cropped images shown in Fig 6A. HSP70 was detected in exosomes derived from conditioned medium of cultured rat BM-MSCs (Lane 1) and exosomes derived from rat serum (Lane 2). The molecular weight of HSP70 is 53–70 kDa. (TIFF) [file pone.0204252.s002.tiff]
